# Deep sequencing of near full-length HIV-1 genomes from plasma identifies circulating subtype C and infrequent occurrence of AC recombinant form in Southern India

**DOI:** 10.1371/journal.pone.0188603

**Published:** 2017-12-08

**Authors:** Shuba Varshini Alampalli, Michael M. Thomson, Raghavan Sampathkumar, Karthi Sivaraman, Anto Jesuraj U. K. J., Chirag Dhar, George D. Souza, Neil Berry, Annapurna Vyakarnam

**Affiliations:** 1 Centre for Infectious Disease Research (CIDR), Indian Institute of Science, Bengaluru, India; 2 Centro Nacional de Microbiología, Instituto de Salud Carlos III, Ctra. Majadahonda-Pozuelo, Majadahonda, Madrid, Spain; 3 Department of Infectious Diseases, St John’s Research Institute, Bengaluru, India; 4 Department of Pulmonary Medicine & Department of Infectious Diseases, St John’s Research Institute, Bengaluru, India; 5 Division of Virology, NIBSC, South Mimms, United Kingdom; 6 Department of Infectious Diseases, King’s College London, London, United Kingdom; University of Liverpool Institute of Infection and Global Health, UNITED KINGDOM

## Abstract

India has the third largest number of HIV-1-infected individuals accounting for approximately 2.1 million people, with a predominance of circulating subtype C strains and a low prevalence of subtype A and A1C and BC recombinant forms, identified over the past two decades. Recovery of near full-length HIV-1 genomes from a plasma source coupled with advances in next generation sequencing (NGS) technologies and development of universal methods for amplifying whole genomes of HIV-1 circulating in a target geography or population provides the opportunity for a detailed analysis of HIV-1 strain identification, evolution and dynamics. Here we describe the development and implementation of approaches for HIV-1 NGS analysis in a southern Indian cohort. Plasma samples (*n* = 20) were obtained from HIV-1-confirmed individuals living in and around the city of Bengaluru. Near full-length genome recovery was obtained for 9 Indian HIV-1 patients, with recovery of full-length *gag* and *env* genes for 10 and 2 additional subjects, respectively. Phylogenetic analyses indicate the majority of sequences to be represented by subtype C viruses branching within a monophyletic clade, comprising viruses from India, Nepal, Myanmar and China and closely related to a southern African cluster, with a low prevalence of the A1C recombinant form also present. Development of algorithms for bespoke recovery and analysis at a local level will further aid clinical management of HIV-1 infected Indian subjects and delineate the progress of the HIV-1 pandemic in this and other geographical regions.

## Introduction

Characterising the genetic composition of HIV-1 circulating in different geographical locales represents a significant undertaking, with implications for vaccine design, treatment efficacy regimes and understanding the molecular epidemiology of transmitted variants in target populations, with group M subtypes (A-K) and circulating recombinant forms (CRFs) comprising the majority of the genotypes in global circulation. HIV-1 clade C infections represent approximately half of the total HIV-1 infections worldwide, being the majority variant in Southern Africa and the Indian subcontinent [1]. HIV-1 clade C therefore represents a significant target for vaccine efforts. We were interested in determining the specific variants of HIV-1 clade C circulating in a cohort of HIV-1-infected individuals in Bengaluru, southern India, using deep sequencing approaches. Previously, whole genome recovery of HIV-1 RNA from plasma using a pan-HIV-1 amplification strategy and next generation sequencing (NGS) and bioinformatics analyses has been reported [2–4], providing fresh insight on HIV-1 variation by taking into account many under-represented HIV-1 variants and recombinant forms in a given host.

In this study, we describe modifications of the whole genome amplification protocol described by Gall *et al* [4] applied to clinically-relevant plasma viruses and a bioinformatics framework in the form of a bespoke pipeline, to amplify HIV-1 genomes from Indian clinical isolates and assemble them reliably post-NGS sequencing. Further, we describe a phylogenetic analysis of these Indian HIV-1 sequences using this pipeline for the first time, as either whole genome sequences or *gag* genes to determine the relationship with the diversity of HIV-1 clade C infections worldwide.

## Materials and methods

### Clinical details

This study was conducted according to the principles expressed in the Declaration of Helsinki. This study was approved by the Ethical Review Committee of St. John’s Medical College Hospital, Bengaluru, India (Ref no: 55/2015). Patients were included in this study after obtaining written consent. Relevant clinical information was documented in a pro forma and 30 ml of EDTA-anticoagulated peripheral blood was collected by venipuncture. A total of twenty subjects confirmed HIV seropositive (by the standard HIV I & II ELISA test and western blot) with known CD4^+^ T-cell counts and plasma HIV viral loads (Roche diagnostics, Germany) were studied (see Table 1). Sixteen out of twenty subjects with no prior history of treatment with antiretroviral drugs [antiretroviral treatment (ART)-naïve] or post-exposure prophylaxis were included and were confirmed to be TB negative. Four out of the twenty HIV positive subjects (SHE001, SC007, SC019 and SC062) were diagnosed with pulmonary and/or extrapulmonary tuberculosis (TB). A diagnosis of pulmonary TB (SHE001) was ascertained by sputum smear microscopy and culture. Standard smear grading of 1+, 2+ and 3+ was used to ascertain the bacterial burden. Smear-negative cases of pulmonary TB cases were diagnosed and classified by the treating clinician from plain chest radiographs as per the Revised National Tuberculosis Control Program (RNTCP) standards. A diagnosis of extra-pulmonary TB (SC007, SC019 and SC062) was established from tissue specimens by Ziehl-Neelsen staining for detection of acid-fast bacilli in tissue samples (obtained as surgical specimens/biopsies/fine-needle aspiration specimens). The RNTCP-approved Nucleic-Acid Amplification Test (NAAT) GeneXpert MTB/Rif of tissue sample was used for confirmation of this diagnosis. Subjects SC007 and SC019 had received treatment for either TB and /or HIV. SC007 was treated for TB (Anti-Tubercular/Tuberculosis Therapy/Treatment (ATT) but ART-naïve) for 52 days, whereas SC019 had completed 6 months of treatment for both TB and HIV prior to blood collection for this study. Of the 20 samples studied, 65% were male; the median age was 33 years (range 24–51); median CD4^+^ T-cell count at the time of study was 463.5 cells/mm^3^ (range 99–853); and the median viral load was 103,300 copies/ml (range 5,978–3,330,000 copies/ml). The correlation between CD4^+^ T-cell count and viral load for each sample is depicted in Figure A in S1 File.

**Table 1:** Clinical data.

S. No.	Sample	Age	Sex	Patient Category and Therapy	Type and site of TB	Date of viral RNA isolation	CD4 count (cells/mm^3^)	Viral Load (copies/ml)
1	SHE001	45	M	HIV+TB+ (Treatment Naïve)	Pulmonary TB, suspected intestinal TB	16-04-2015	773	138000
2	SC007	26	M	HIV+TB+ (ATT 52 days and ART-naïve)	Extra pulmonary TB, TB lymphadenitis	05-05-2015	510	725000
3	SC008	49	M	HIV+ ART-naïve	None	20-05-2015	389	2980000
4	SC012	34	F	HIV+ ART-naïve	None	25-05-2015	582	14100
5	SC013	30	F	HIV+ ART-naïve	None	28-05-2015	510	120000
6	SC015	30	M	HIV+ ART-naïve	None	29-05-2015	415	63000
7	SC017	45	M	HIV+ ART-naïve	None	01-06-2015	475	108000
8	SC018	31	M	HIV+ ART-naïve	None	02-06-2015	382	9010
9	SC019	34	M	HIV+TB+ (Treated for both for 6 months)	Extra pulmonary TB, Abdominal TB	06-02-2015	267	347000
10	SC022	42	F	HIV+ ART-naïve	None	08-06-2015	853	39900
11	SC023	49	M	HIV+ ART-naïve	None	08-06-2015	583	98600
12	SC061	30	F	HIV+ ART-naïve	None	31-08-2015	452	80900
13	SC062	51	M	HIV+TB+ (Treatment Naïve)	Extra pulmonary TB, Lymph node	03-09-2015	167	752000
14	SC063	28	F	HIV+ ART-naïve	None	07-09-2015	158	81600
15	SC064	35	M	HIV+ ART-naïve	None	07-09-2015	109	3330000
16	SC065	24	M	HIV+ ART-naïve	None	08-09-2015	497	10800
17	SC072	32	M	HIV+ ART-naïve	None	29-09-2015	230	5970
18	SC073	27	M	HIV+ ART-naïve	None	29-09-2015	581	131000
19	SC078	32	F	HIV+ ART-naïve	None	06-10-2015	755	13300
20	SC085	42	F	HIV+ ART-naïve	None	03-11-2015	99	368000

### Viral RNA isolation

Viral RNA was purified from 1 ml of EDTA-treated plasma using the QIAamp viral RNA mini-kits (Qiagen, Cat # 52906) as per manufacturer protocol with some modifications. Instead of the recommended 140 μl, 1 ml of EDTA-treated plasma was processed in a single QIAamp Mini column and RNA was eluted in two batches of 40 μl of elution buffer each. 10 μl aliquots of purified plasma RNA were stored at -20°C for immediate use (less than 2 hours); long-term storage of RNA was at -80°C.

### Primer design and one-step RT-PCR

The strategy adopted was to develop the pan-HIV-1 primer approach for the putative amplification of HIV-1 genomes of all major groups (M, N and O), their subtypes and recombinants as previously reported [4]. Initial analysis of field samples from the Indian cohort suggested modifications of primers to improve amplification efficiency and coverage of the *gag-pol* region were required. Further modifications were introduced based on consensus C-clade genome sequences retrieved from the Los Alamos HIV Sequence Database and an extended amplicon strategy adopted (summarized in Figure B in S1 File). Using the primers shown in Table 2, one-step RT-PCRs were performed to derive overlapping amplicons of 1.9 kb, 1.1 kb, 1.4 kb, 1.7 kb, 3 kb, and 3.5 kb using SuperScript III One-Step RT-PCR with Platinum *Taq* DNA High Fidelity Polymerase kits (Invitrogen, Cat # 12574–035). Each 25 μl reaction mixture contained 12.5 μl reaction mix (2 x), 4.5 μl RNase-free water, 1 μl each of each primer (conc. 20 nmol/μl), 1 μl SuperScript III RT/ Platinum Taq High Fidelity mix and 5 μl of template RNA. Cycling conditions were 50°C for 30 min; 94°C for 2 min; 35 cycles of 94°C for 15 sec, 58°C for 30 sec, and 68°C for 4 min 30 sec; and, finally, 68°C for 10 min. Amplicon quality was verified by agarose gel electrophoresis.

**Table 2:** Primers used in this study.

Amplicon Name	Primer Sequence	Coordinates on HXB2 genome	Reference
Amp1	Pan-HIV-1F: AGCCYGGGAGCTCTCTG Pan-HIV-1R: CCTCCAATTCCYCCTATCATTTT	26–1953 (1928 bp)	Gall et al., JCM 2012
Amp2 (1F&1R)	HIV-A2-SE-F1: AgTATgggCAAgCAgggAgCT HIV-A2-SE-R1: TgTCCTTCCTTTCCACATTTCC	891-2051(1160 bp; overlap with Amp1: 1062 bp)	This study
Amp3 (2F&3R)	HIV-A2-SE-F2: gATgACAgCATgTCAgggAgT HIV-A2-SE-R3: TATAggCTgTACTgTCCATTA	1827–3281 (1454 bp; overlap with Amp2: 224 bp)	This study
Amp4 (4F&4R)	HIV-A2-SE-F4: CTTCCACAgggATggAAggAT HIV-A2-SE-R4: CTgCCATTTgTACTgCTgTCTT	2994–4767 (1773 bp; overlap with Amp3: 287 bp)	This study
Amp5	Pan-HIV-3F: TTAAAAGAAAAGGGGGGATTGGG Pan-HIV-3R: TGGCYTGTACCGTCAGCG	4329–7394 (3066 bp; overlap with Amp4: 438 bp)	Gall et al., JCM 2012
Amp6	Pan-HIV-4F: CCTATGGCAGGAAGAAGCG Pan-HIV-4R: CTTWTATGCAGCWTCTGAGGG	5513–9063 (3551 bp; overlap with Amp5:1881 bp)	Gall et al., JCM 2012

### Illumina sequencing

Massive parallel sequencing of the pooled amplicons was carried out on an Illumina NextSeq 500 analyser. Library preparation was performed at Genotypic Technology’s Genomics facility following Nextera XT DNA Library Preparation protocol (Cat #FC-131-1024). 1 ng of Qubit-quantified genomic DNA was tagmented (fragmented and tagged) using Amplicon Tagment Mix provided in the Nextera XT Kit. The adapter tagged DNA was subjected to 12 cycles of indexing-PCR [72°C for 3 min followed by denaturation at 95°C for 30 sec, cycling (95°C for 10 sec, 55°C for 30 sec, 72°C for 30 sec) and 72°C for 5 min] to enrich the adapter-tagged fragments. The PCR product was purified using HighPrep beads (Magbio Genomics, #AC-60050). Quantification and size distribution of the prepared libraries were determined using Qubit fluorometer and the Agilent D1000 TapeStation respectively according to the manufacturer’s instructions.

### Bioinformatics

#### QC and genome assembly

Raw reads generated from the Illumina machine were subjected to quality assessment using FastQC [5] and Trimmomatic v0.36 [6]. Based on per base quality score and k-mer presence, sequences were trimmed 10 bases at the 5’-end and five bases at 3’-end. Trimmed reads were mapped using bowtie2 [7] (default parameters) onto 2870 complete HIV-1 genomes to identify the closest reference. HIV-1 mapped reads were extracted and *de novo* assembled using IVA (Iterative Virus Assembler) [8]. IVA-assembled contigs were used as input for PRICE (Paired-Read Iterative Contig Extension) [9], with default options. PRICE uses incomplete assemblies of the clinical isolate genome and extends the contigs to fill the gaps iteratively. We ran PRICE for 10 iterations, choosing all contigs above 1000 bases at each cycle as templates for the next cycle. In nine cases, we obtained near complete genome assemblies (size > 8.5 kb) using this strategy and the remaining eleven cases could be assembled to obtain the *gag* gene owing to inadequate coverage of reads. In two cases, we assembled both *gag* and *env* genes but did not have enough read support to complete the whole genome. The genomes were made into their own consensus sequence using samtools [10] and bcftools [11].

#### Phylogenetic analyses

HIV-1 reference sequences were downloaded from the Los Alamos HIV Sequence Database [12] for phylogenetic analyses. Multiple sequence alignment was performed using MUSCLE [13] (default parameters) and phylogenetic trees were generated via maximum likelihood (ML) under the general time-reversible with gamma-distributed rate heterogeneity among sites (GTR+Γ) evolutionary model using RAxML v8.2.10 [14]. Trees were visualized with MEGA 7 [15]. The presence of intersubtype recombination was initially checked with Recombination Identification Program (RIP) [16], with subsequent analysis of the mosaic structure with the bootscanning method implemented in Simplot v3.5.1 [17]. For SNP-based phylogenetic tree, the HIV-1 specific filter reads for each sample were trimmed (adaptor/transposon sequence, 5’ < Q10 and 3’ <Q20) and clipped for an average quality score of 20 using Trimmomatic v0.36. QC-ed reads were mapped using Bowtie2 and duplicates marked using Picard MarkDuplicates [18]. SNP/InDels were saved in VCF format using samtools and bcftools. In order to include multiple reference sequences for a SNP-based phylogenetic tree, multiple sequence alignment among the reference was carried out with MUSCLE and corresponding bases (indicated as SNP/InDel by vcftools [19]) against each reference were extracted and concatenated using in-house R-scripts. The SNPs and InDels were concatenated for each sample following the same order of sequences in each case.

## Results

### Amplicon recovery and protocol optimization

Adaptation of the 4-amplicon strategy to recover near full-length HIV-1 genomes to a 6-amplicon strategy (Figure B in S1 File) for large-scale HIV-1 genome sequencing improved recovery of HIV-1 gene fragments from the Bengaluru cohort. While providing enhanced recovery of viral strains circulating in this population, amplification efficiency remained variable with some isolates, which appeared independent of baseline viral load status. Amplicons for each primer-pair, pooled in equimolar concentrations based upon NanoDrop readings, resulted in more than 1 million reads for each sample (minimum 30,000 reads per amplicon).

### Initial de novo assembly and extension

In order to assemble a viable HIV-1 genome and enhance the potentially unique nature of each clinical isolate, we followed a two-step process involving *de novo* assembly using IVA (step 1) and PRICE (step 2). Extracted reads were mapped onto a consensus C-clade genome with the resulting read coverage for each sample shown in Table 3. SC012, SC018, SC019, SC023, SC061, SC063, SC064, SC065, SC072, SC073 and SC078 were analyzed across *gag* as a result of low coverage downstream of the *pol* gene.

**Table 3:** Summary of NGS results for each viral isolate.

Whole Genome												
							Read Count for each primer used in this study					
Sl.No.	Sample ID	Total No. of paired-end reads	Percentage of HIV-1 specific reads	Contigs After PRICE		Length of the contig	Amp1	Amp2	Amp3	Amp4	Amp5	Amp6
1	SC007	12,048,590	45.86%	1		9041	1,081,560	839,188	2,560,787	2,406,795	38,537	17,069
2	SC008	3,456,568	16.75%	1		9084	82,876	58,111	193,065	312,744	34,558	16,670
3	SHE001	3,168,528	28.06%	1		9114	111,893	85,609	321,476	595,475	15,005	4,469
4	SC013	2,259,720	29.48%	1		9187	439,695	399,894	161,808	64,259	93,514	16,011
5	SC015	6,464,458	2.37%	1		9122	75,807	64,536	22,762	5,293	11,775	4,483
6	SC017	6,119,426	3.02%	1		9043	83,658	79,833	52,115	68,269	1,665	443
7	SC022	6,891,246	8.60%	1		8940	327,410	309,199	97,578	125,710	26,937	6,228
8	SC062	6,649,608	25.91%	1		9073	894,930	846,293	354,433	258,557	55,657	29,616
9	SC085	1,558,952	14.98%	1		9117	127,991	110,737	113,634	28,640	34,927	9,489
Partial Genome												
							Read Count for each primer used in this study					
Sl.No.	Sample ID	Total No. of paired-end reads	Percentage of HIV-1 specific reads		Contigs After PRICE	Length of the contig	Amp1	Amp2	Amp3	Amp4	Amp5	Amp6
1	SC012	223,832	1.57%		1	1839	2,720	2,673	352	0	0	0
2	SC018	305,190	34.23%		1	2924	62,556	58,685	9,208	64	198	67
3	SC019	6,233,546	15.60%		2	4959;3621	602,209	569,824	112,558	22,808	34,062	10,188
4	SC023	6,185,790	9.91%		2	4841;4384	481,739	431,574	75,507	6,475	7,109	8,795
5	SC061	300,540	52.14%		2	3076;1324	88,149	81,933	14,422	203	360	1,014
6	SC063	1,720,104	2.33%		3	2948;2354;1919	47,377	42,021	7,144	788	515	1,394
7	SC064	168,998	63.92%		2	3592;3297	61,325	56,149	13,412	1,328	3,570	1,346
8	SC065	1,869,004	2.62%		1	1978	56,656	52,643	2,304	2	160	221
9	SC072	156,858	0.68%		1	2748	390	300	66	13	0	0
10	SC073	297,644	15.23%		1	2150	32,580	32,123	4,005	2	102	30

IVA uses an overlap consensus layout along with iteratively aligning reads to contigs so as to minimise sequencing errors and rare variations within the quasispecies. PRICE utilises the preassembled contigs from IVA and extends iteratively, until sufficiently long contigs are produced and gaps filled. In our runs, use of PRICE allowed us to merge multiple kilobase-sized contigs (Table 3) into a single genome-size assembly (>8500 bases) for nine strains.

### Phylogenetic analysis of the clinical samples

Of the 20 Indian clinical samples, 9 yielded near full-length genomes (NFLG), with 10 further samples assembled across the complete *gag* gene. Sample SC078 had very few HIV-1 specific reads and could not be assembled across the complete *gag* gene and was therefore removed from further analyses.

**Fig 1:**
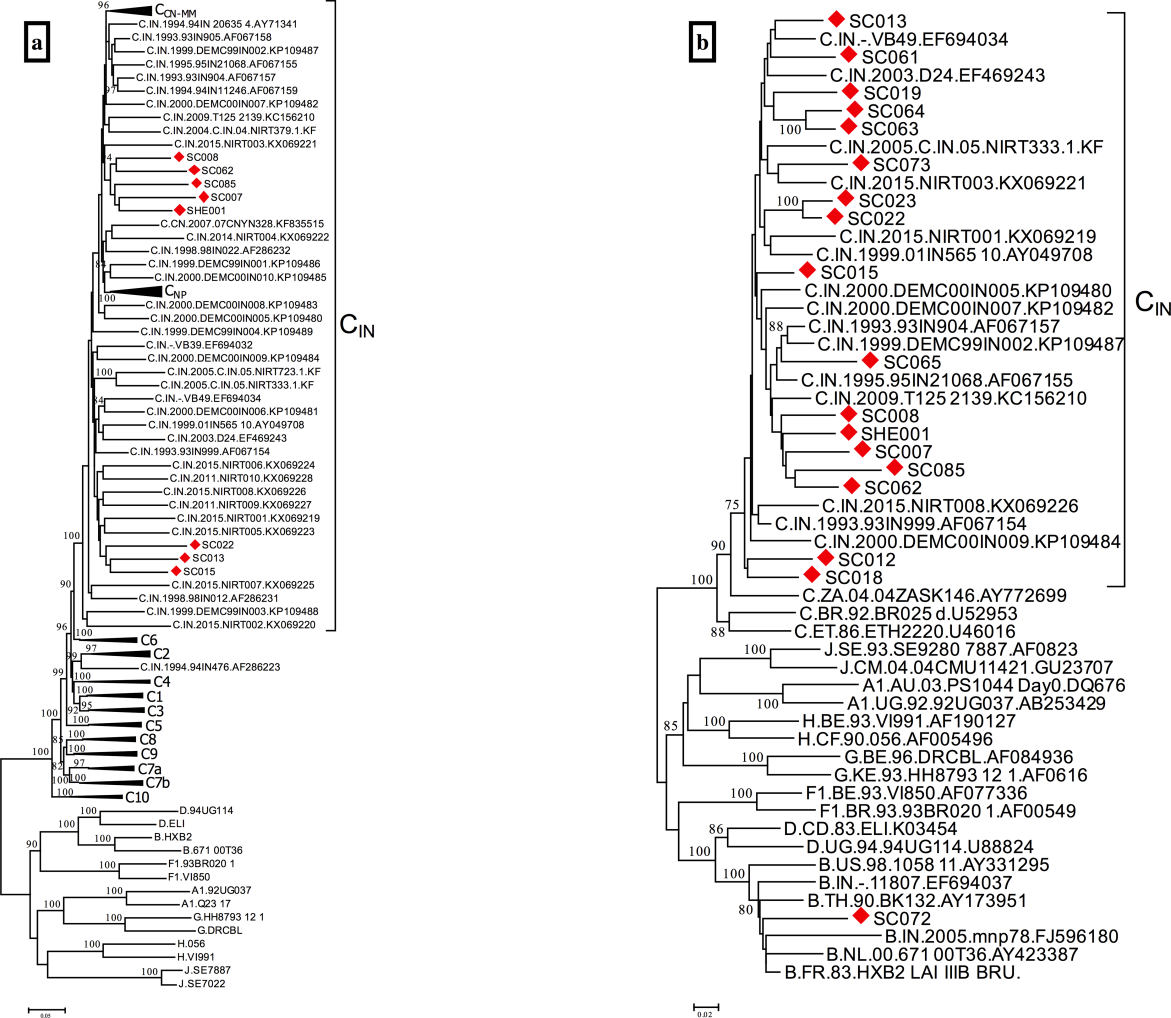
Maximum likelihood phylogenetic trees of HIV-1 sequences from Bengaluru. Sequences from Bengaluru obtained in this study are labelled with red diamonds. Only bootstrap values ≥70% are shown. (a) Phylogenetic tree of NFLG sequences of subtype C. All NFLG subtype C sequences from Asia available at the Los Alamos HIV Sequence Database are included in the analysis. References of African subtype C clusters C1 through C10 [20] are also included and are shown compressed in triangles, as are clusters comprising sequences from China and Myanmar (C_CN-MM_) and from Nepal (C_NP_) (the last cluster comprising one sequence from India) nested within the Indian clade (C_IN_). (b) Phylogenetic tree of *gag* sequences of subtypes C and B. Fifteen randomly selected subtype C sequences and the only two subtype B sequences from India available at the Los Alamos database were included in the analysis, together with subtype references.

Since recombinant sequences can affect node support values and topologies in a phylogenetic tree, prior to tree construction, the newly obtained sequences were checked for the presence of intersubtype recombination using RIP. These analyses indicated that one virus, SC017, was A1C recombinant and all other sequences were of homogeneous subtypes (subtype C, except SC072 *gag* sequence, which was of subtype B). ML trees were constructed based on either whole-genome (Fig 1A) or *gag* alone (Fig 1B), excluding SC017 from the analyses. Multiple sequence alignments were generated with the HIV-1 subtype references downloaded from the Los Alamos HIV Sequence Database. In order to examine the phylogenetic relationship of the subtype C viruses from Bengaluru with other subtype C viruses from India and other countries, in the whole-genome tree, all subtype C NFLG sequences from India and other Asian countries (China, Myanmar and Nepal) available at the Los Alamos database, as well as references from the 11 subtype C clusters previously identified in Africa [20], were included in the analysis. The ML tree generated with this alignment (Fig 1A) shows that the Bengaluru sequences branch within a strongly supported monophyletic clade comprising all but one Asian subtype C sequences (the only exception being one sequence from India), within which sequences from Nepal, on the one hand, and China and Myanmar on the other, group in respective sub-clusters. The Asian subtype C clade is closely related to the previously identified African C6 cluster, comprising viruses from Botswana and South Africa, which in turn forms part of a supercluster comprising six clusters (C1 through C6) associated with southern Africa [20]. Within the Asian subtype C clade, the Bengaluru sequences failed to group with each other, except two sequences, SC008 and SC062, which joined in a cluster supported by a 94% bootstrap value. In the *gag* tree (Fig 1B), in which 15 other subtype C and the two subtype B full-length *gag* sequences from India available at the Los Alamos database were included, 18 sequences from Bengaluru were of subtype C, branching within the Indian/Asian clade, with only two pairs of sequences (SC022/SC023; SC063/SC064) grouping in well supported clusters, and one was of subtype B. Both viruses sequenced only in *env* were subtype C, branching within the C_IN_ clade, in agreement with the *gag* phylogenetic tree (results not shown).

SNP based phylogenetic tree was constructed in the same way and the orientation of the samples reflects the results of the previous trees. The SNPs and InDels in each sample annotated on consensus subtype-C genome are represented in Figure C in S1 File and many of the variations appear to be in the *env* gene.

**Fig 2:**
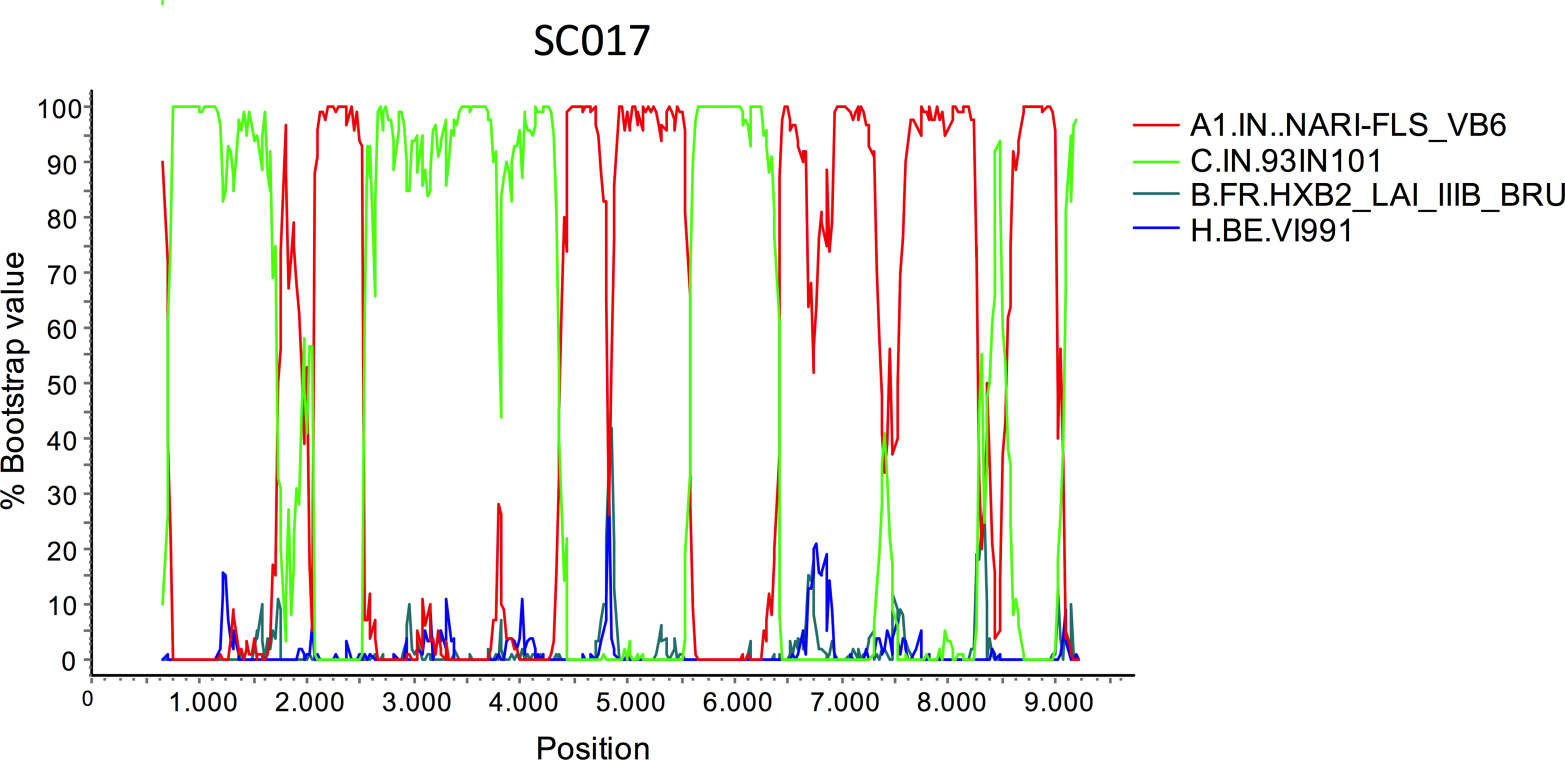
Bootscan analysis of the NFLG of SC017. The horizontal axis represents the position in the HXB2 genome and the vertical axis represents percent bootstrap values supporting clustering with reference sequences. Trees were constructed with the neighbor-joining method, using a window of 250 nt, sliding along the alignment in 20 nt steps.

The mosaic structure of SC017 was analyzed by bootscanning using Indian A1 and C subtype references, together with references of subtypes B and H used as outgroups. The resulting bootscan plot (Fig 2) shows a complex structure with multiple breakpoints along the genome. To examine whether SC017 is related to other Indian A1C recombinant viruses, an ML phylogenetic tree was constructed with all Indian A1C recombinant NFLG sequences (only one per patient) deposited at the Los Alamos database. The tree shows clustering (75% bootstrap support) of SC017 only with one A1C recombinant, 95IN21301 (Figure D in S1 File). However, its mosaic structure differs substantially from that of SC017 (Figure E in S1 File).

NCBI GenBank accession numbers for the sequences reported here are: KY713228 (SC007); KY713229 (SC008); KY713230 (SC013); KY713231 (SC015); KY713232 (SC017); KY713233 (SC022); KY713234 (SC062); KY713235 (SC085); KY713236 (SHE001); KY713237 (*gag* gene SC012); KY713238 (*gag* gene SC018); KY713239 (*gag* gene SC061); KY713240 (*gag* gene SC063); KY713241 (*gag* gene SC064); KY713242 (*gag* gene SC065); KY713243 (*gag* gene SC072); KY713244 (*gag* gene SC073); KY713245 (*gag* gene SC019); KY713246 (*env* gene SC019); KY713247 (*gag* gene SC023); KY713248 (*env* gene SC023).

## Discussion

Here, we describe near full length genome recovery and assembly of HIV-1 from a clinical cohort in southern India using plasma-derived viral RNA, comprising recently replicating viral variants circulating in 20 HIV-1-diagnosed individuals from Bengaluru city. Overlapping amplicons using RT-PCR-based protocols and Illumina NextSeq 150 technology were used to generate *de novo* genome assemblies, optimized to enhance sequencing data/flow-through and to reflect evolution of HIV-1 genomes using phylogenetic analyses. Several universal methods for amplifying and sequencing whole HIV-1 genomes have been reported [2–4, 21–24]. However, we adapted the universal amplification protocol of Gall *et al* [4], where oligonucleotides were originally selected based on the large number of collated HIV-1 sequences in the Los Alamos database minimising amplification bias within highly conserved regions using bioinformatics predictions of sequence fidelity. Originally performed on 10 WHO HIV-1 genotype reference strains and 15 HIV-1 plasma RNA samples of various subtypes and CRFs an overall sensitivity of detection of 3000 HIV-1 RNA copies/ml was achieved. However, direct application of the universal primers appeared suboptimal for many field strains circulating in India, with plasma virus representing the primary source of viral RNA and we were unable to improve on the 3000 RNA copy number detection threshold; an overall 70% amplification efficiency success rate was, however, consistent with previous findings [2]. Interestingly, the inability to amplify from a proportion of Indian HIV-1 strains appeared independent of viral load even with re-design of primers, for example, to the *gag/pol* region making further refinement and re-design of these protocols inevitable. This is perhaps due to lack of documented circulating strains from South India, which can be used as a template for more efficient primer design and our data emphasizes significant differences in the *gag-pol* region of the circulating strains compared to the available reference genomes.

While Illumina sequencing is a stochastic process which may not attain equal coverage of all amplicons in a given pool, this could potentially be overcome by preparing individual libraries for each amplicon of every sample. However, the significant associated cost escalation precluded using this option. At least a million reads of a single library were generated per sample (comprising an equimolar pool of all 6 amplicons), resulting in ~30,000 reads per amplicon which represented a mathematical minimum requirement for downstream *de novo* genome assembly. In addition, *de novo* assemblies are not robust in assembling whole genomes from short, paired-end data with inherent gaps in sequence coverage. Mindful of these limitations, in an Indian clade C dominant population, we manually assessed read quality, assembly construction and number of iterations during assembly extension for each sample, rather than use a standard reference-based assembly approach.

The data generated extends our knowledge of HIV-1 strain characterization of viruses circulating in India reported since the 1990s, confirming that in addition to the largely subtype C predominance, subtypes A1 and B are also circulating as minor clades giving rise to A1C and BC recombinant forms [25–35]. Although analyses have usually been restricted to single genes, typically *gag*, *pol*, or *env*, some authors have reported sequencing of full-length proviral DNA of HIV-1 in India from cultured peripheral blood mononuclear cells [17,33,35–38], representative of both archived and replicating viral genomes. Reports of HIV-1 whole genome sequencing from plasma RNA, to identify viruses that are currently replicating and circulating in India, is however limited to one publication [2], in which sequences from 10 field HIV-1 samples genotypically confirmed to be subtype C strains were reported.

Phylogenetic analyses of partial or full-length genomes have shown that the great majority of HIV-1 subtype C strains from India group in a monophyletic clade [17,25,26,30,36,38–41], designated C_IN_ [26], which, according to phylodynamic analyses, originated in the 1970s [41–43], although sporadic cases of subtype C viruses branching outside of the C_IN_ clade have been reported [6,30,36,41]. Analyses of relationships with viruses from other countries indicate that the African viruses most closely related to C_IN_ are from the southern part of the continent [36,41], and that subtype C strains circulating in China [36,42,44], Myanmar [42], Nepal [45,46] and Bangladesh [47] derive from the Indian clade. Our ML phylogenetic trees confirm the monophyly of most Indian subtype C viruses, with all 8 NFLG and 18 *gag* subtype C viruses from Bengaluru branching within the C_IN_ clade, interspersed among other Indian viruses (Fig 1). Additionally, for the first time we examine the relationship of the C_IN_ clade with the previously identified African subtype C clusters [20], finding that it is closely related to a cluster (C6) comprising viruses from Botswana and South Africa (Fig 1A), which in turn groups in a supercluster (C1-C6) associated to Southern African countries, thus confirming previous reports indicating a close relationship between Indian and Southern African subtype C viruses.

Of the 18 viruses from Bengaluru characterized in the NFLG or *gag* or *env* genes, only two were of non-subtype C genetic forms: one A1C recombinant (SC017), characterized in the NFLG (Fig 2), and one of subtype B (SC072), characterized in *gag* (Fig 1B). These results are consistent with previous studies reporting subtype B and A1C viruses to be minor components of the Indian HIV-1 epidemic [17,27,28,31–33]. SC017 exhibited a complex mosaic structure, with multiple breakpoints (Fig 2). In a phylogenetic tree, it appeared to be related to another A1C recombinant from India (Figure D in S1 File), although their mosaic structures were clearly different (Figure E in S1 File), implying that SC017 represents a unique recombinant form, as do other A1C recombinants characterized in India, which fail to cluster with each other (Figure D in S1 File).

The extent of genetic mixing and its detailed nature in the context of the widespread distribution of HIV-1 subtype C in India will only become apparent by more systematic and detailed sequencing studies underscoring the utility of deep sequencing approaches to identify changes in the balance of epidemiologically relevant strains and the appearance of novel recombinants. These are most likely to be detected by direct sequence recovery from plasma samples, taken as evidence of productive, systemic infection and recent replication events in an individual where novel variants and recombinant forms will be detectable. Further analysis of the nine whole genomes and historical database isolates suggests limited evolution over time where the virus appears well-adapted to its host. Similar clustering patterns obtained with whole genome or *gag* gene suggests that overall recombination frequencies are also low. However, greater confidence in depicting the emergence of low frequency variants from whole genome recovery, (e.g. SC017, HIV-1 A1C) will be attained with the ability to fully map whole mosaic genomes. These will be further illuminated by refining and developing the types of approach described in this report, and elsewhere, given the urgent need for an effective HIV vaccine which is particularly able to target subtype C infections.

## Conclusions

In conclusion, our data further indicate that Indian HIV-1 whole genome sequences converge to form a monophyletic lineage of subtype C, closely related to a southern African lineage, with sporadic cases of A1C recombinant forms exhibiting independent origins. While refinement of amplification protocols, sequencing platforms and bioinformatics tools for analysis of this kind of data are ongoing and likely to supersede the current study, application of techniques and approaches described here will benefit both the clinical management of HIV-1 patients, but will further allow a more precise description of molecular epidemiological trends and direction in specific geographical locales. Understanding complex interactions with coincident infections, such as tuberculosis or viral infections, will be facilitated by these types of approach. Such studies will benefit not only treatment approaches and regimens, but provide clearer data for vaccine design approaches when they become available.

## Supporting information

S1 File A) CD4^+^ T-cell count and viral load correlation of clinical samples. B) Primer redesigning for amplicon recovery of samples involved in this study. C) SNPs and InDels annotated on consensus C genome for each sample. D) Maximum likelihood phylogenetic tree of NFLG sequences of SC017 and other A1C recombinant viruses from India. E) Bootscan analysis of the NFLG of 95IN21301.
(PDF)
