# Supplementary material for: Deep sequencing of near full-length HIV-1 genomes from plasma identifies circulating subtype C and infrequent occurrence of AC recombinant form in Southern India
Source: PLoS One. 2017 Dec 8;12(12):e0188603. doi: 10.1371/journal.pone.0188603 (PMC5722309; doi:10.1371/journal.pone.0188603)
Supplement: S1 File — A) CD4+ T-cell count and viral load correlation of clinical samples. B) Primer redesigning for amplicon recovery of samples involved in this study. C) SNPs and InDels annotated on consensus C genome for each sample. D) Maximum likelihood phylogenetic tree of NFLG sequences of SC017 and other A1C recombinant viruses from India. E) Bootscan analysis of the NFLG of 95IN21301. (PDF) [file pone.0188603.s001.pdf]

# Deep sequencing of near full-length HIV-1 genomes from plasma identifies circulating subtype C and infrequent occurrence of AC recombinant form in Southern India.

Shuba Varshini Alampalli, Michael M. Thomson, Raghavan Sampathkumar, Karthi Sivaraman, Anto Jesuraj UK J, Chirag Dhar, George D Souza, Neil Berry & Annapurna Vyakarnam

**Supplementary Figures**


| Contents | Page Number |
|---|---|
| Figure A: CD4 count and viral load correlation of clinical samples. | 2 |
| Figure B: Primer redesigning for amplicon recovery of samples involved in this study. | 3 |
| Figure C: SNPs and InDels annotated on consensus C genome for each sample. | 4 |
| Figure D: Maximum likelihood phylogenetic tree of NFLG sequences of SC017 and other A1C recombinant viruses from India. | 6 |
| Figure E: Bootscan analysis of the NFLG of 95IN21301. | 8 |

**Figure A: CD4 count and viral load correlation of clinical samples.**

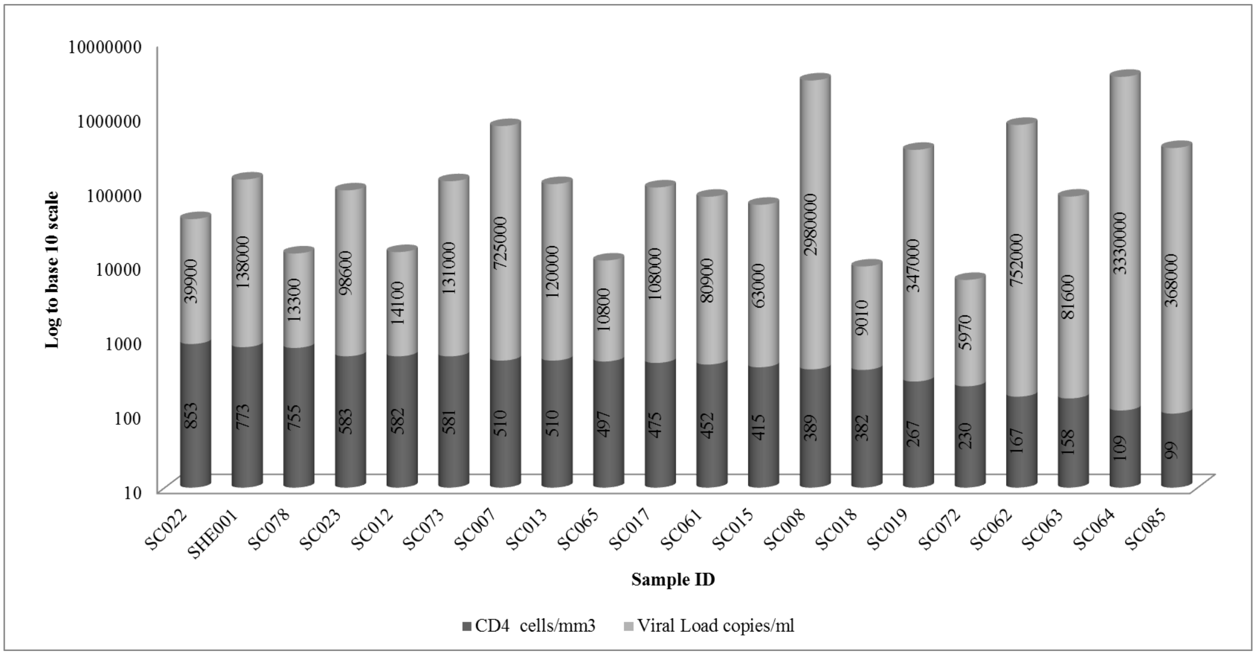


CD4 count is represented in dark grey while the viral load is represented in light grey. The correlation is seen as decreasing CD4 count on Log to base 10 scale.

**Figure B: Primer redesigning for amplicon recovery of samples involved in this study.**

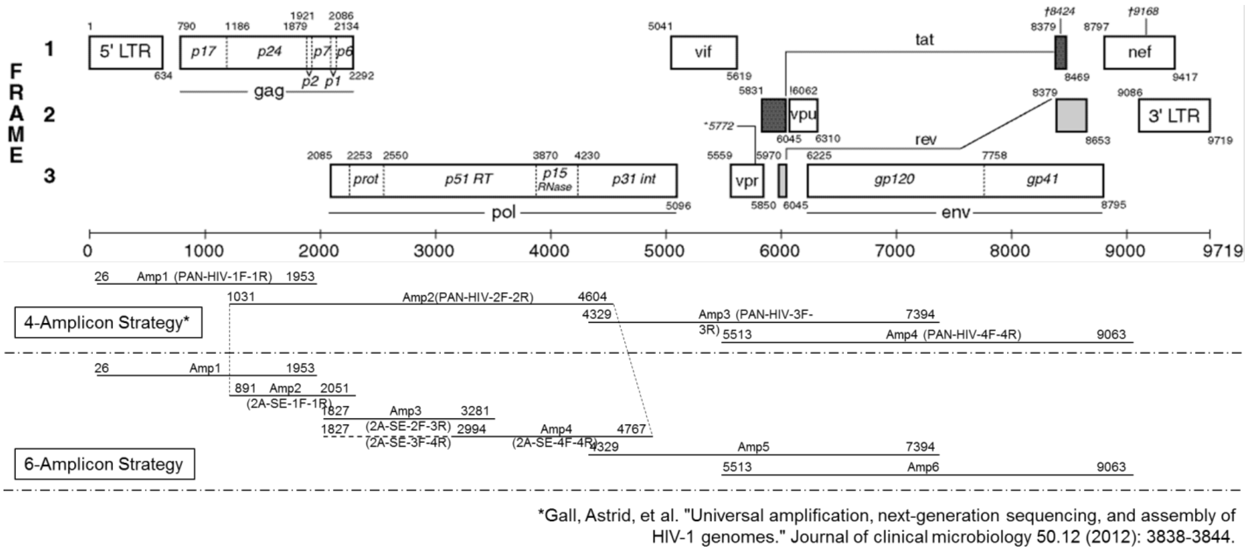


The 4-Amplicon strategy was adopted from Gall et al. [ref 4]. Vertical dotted lines indicate regions where alternative primers to amplify HIV-1 from Indian clinical samples were applied.

**Figure C: SNPs and InDels annotated on consensus C genome for each sample.**

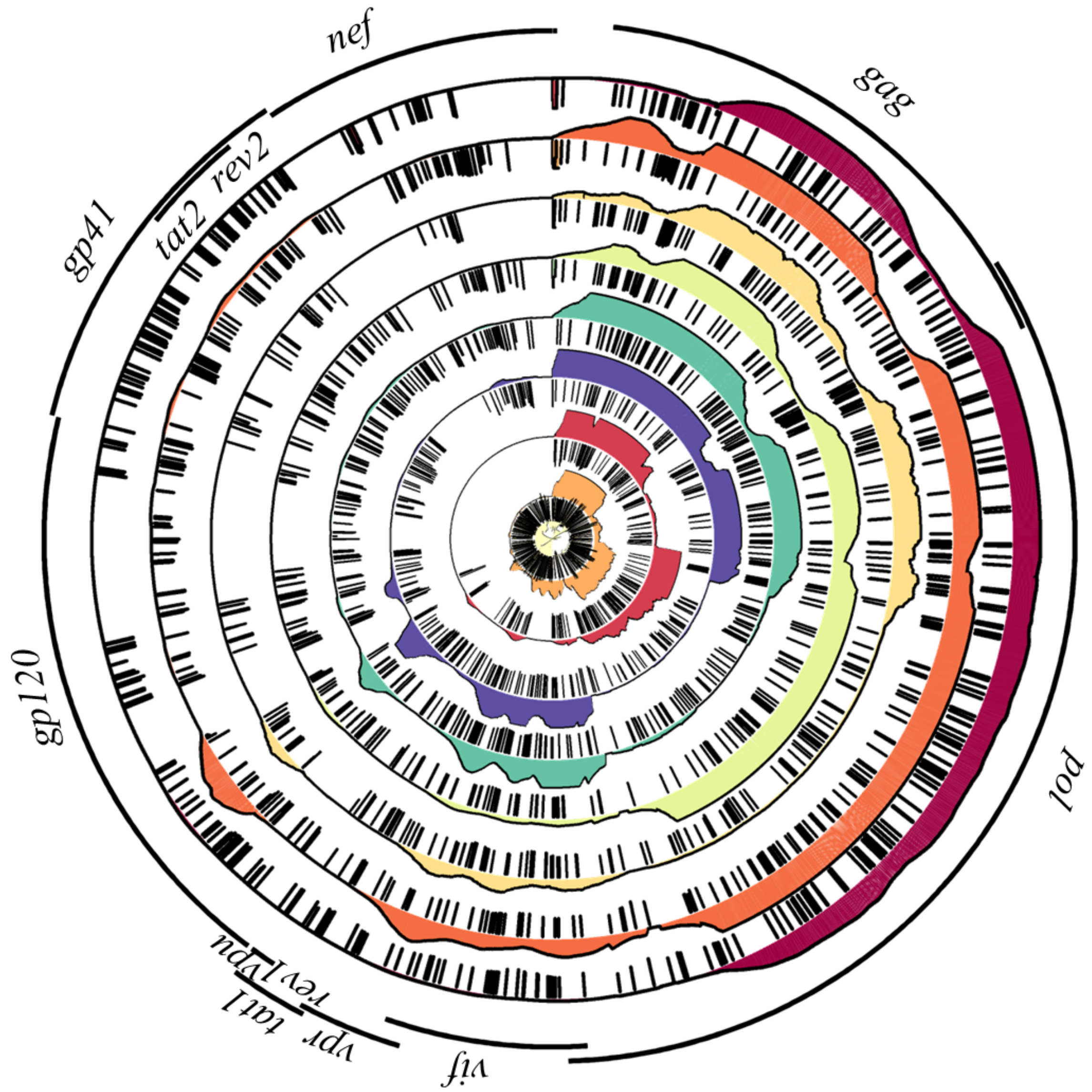


Intra-patient occurrence of SNPs and InDels annotated against the consensus clade C genome using CIRCOS. The circos plot has nine tracks indicating nine whole genomes constructed in this study. There are two histograms plotted for each track. The histogram oriented outward is the coverage (percentage of number of reads aligned to the base by total number of reads mapped to consensus C genome) of each base in consensus C genome. The histogram oriented inward is

the SNP or InDel supported by the fraction of reads mapped at that base/region on consensus C genome.

**Figure D: Maximum likelihood phylogenetic tree of NFLG sequences of SC017 and other A1C recombinant viruses from India.**

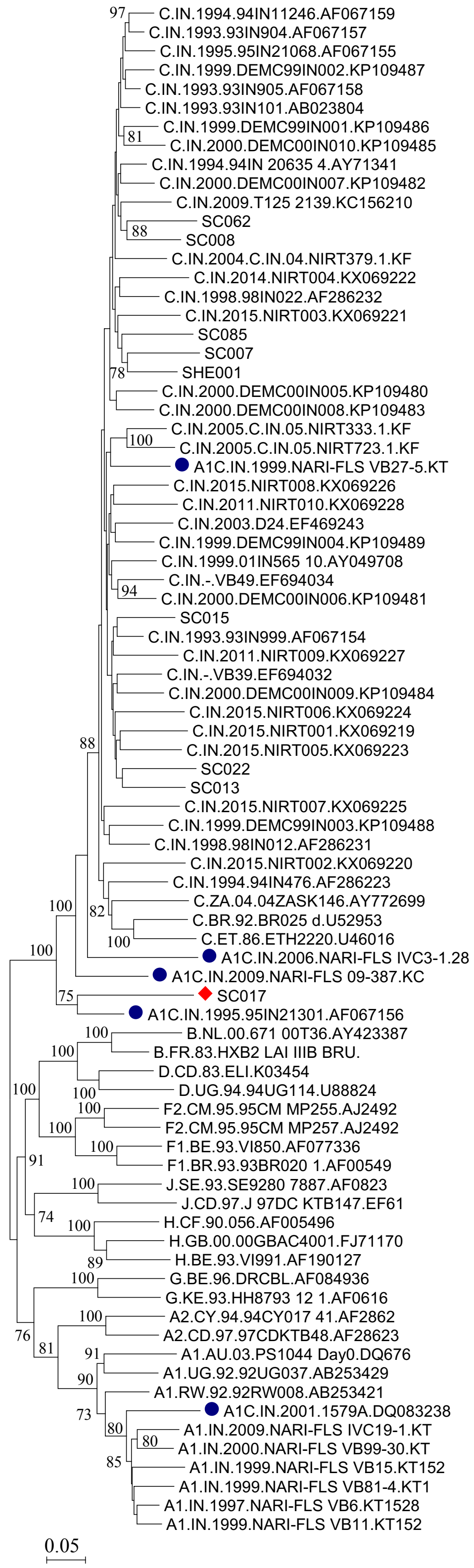

A1 and C subtype sequences from India are also included in the analysis. Only bootstrap values ≥70% are shown. SC017 is labeled with a red diamond and the other A1C viruses from India are labeled with blue circles.

**Figure E: Bootscan analysis of the NFLG of 95IN21301.**

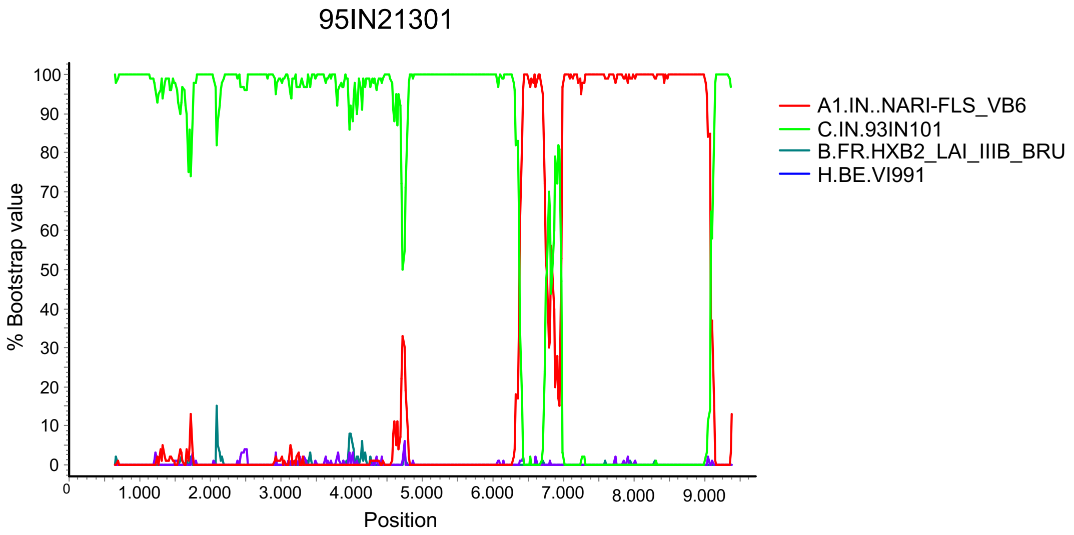


The horizontal axis represents the position in the HXB2 genome and the vertical axis represents percent bootstrap values supporting clustering with reference sequences. Trees were constructed with the neighbor-joining method, using a window of 250 nt, sliding along the alignment in 20 nt steps.
